# Analysis on the healing of gastrointestinal ulceration by using Hemospray

**DOI:** 10.1038/s41598-021-98664-8

**Published:** 2021-09-24

**Authors:** Christoph R. Werner, Lena Brücklmeier, Thomas Kratt, Nisar P. Malek, Bence Sipos, Dörte Wichmann, Martin Götz

**Affiliations:** 1 Department of Gastroenterology, Gastrointestinal Oncology, Hepatology, Infectiology, and Geriatrics, University Hospital Tübingen, 72076 Tübingen, Germany; 2 Department of Surgery, Kliniken Böblingen, 71032 Böblingen, Germany; 3 Department of Internal Medicine, Spital Limmattal, 8952 Zürich, Switzerland; 4 BAG für Pathologie und Molekularpathologie, 70176 Stuttgart, Germany; 5 Department of General, Visceral and Transplantation Surgery, University Hospital of Tübingen, Hoppe-Seyler-Str. 3, 72076 Tübingen, Germany; 6 Department of Gastroenterology and Oncology, Kliniken Böblingen, Böblingen, Germany

**Keywords:** Gastroenterology, Medical research

## Abstract

Healing of gastrointestinal ulcers after Hemospray application was reported in literature. The pathophysiological mechanism of action of hemostatic powders is not elucidated so far. A prospective animal model was performed to evaluate the effect of Hemospray application on the healing process of artificially induced ulcers of the upper and lower gastrointestinal tract. In 10 pigs, 20 ulcers were created in each the upper and the lower gastrointestinal tract by endoscopic mucosal resection. 50% of the pigs were immediately treated with Hemospray application, the others were not treated. Ulcer size was measured endoscopically on day 0, 2, and 7. On day 7 the ulcers were histopathological evaluated for capillary ingrowth and the thickness of the collagen layer. After 7 days the sizes of the ulcers decreased significantly (stomach: − 22.8% with Hemospray application, − 19% without Hemospray application; rectum: − 50.8% with Hemospray application, − 49.5% without Hemospray application; *p* = 0.005–0.037), but without significant difference between both groups. This study shows no significant effect of the hemostatic powder Hemospray on ulcer healing in the upper and lower gastrointestinal tract compared with untreated controls, neither harmful nor beneficial. However, some trends merit further trials in patients and may indicate a possible mechanism of accelerated mucosal healing.

## Introduction

The most frequent sources of non-variceal bleeding in the upper gastrointestinal (GI) tract are ulcers of the stomach and duodenum. The incidence of Helicobacter pylori associated ulcers in the upper GI tract is declining, while other pathogenic causes as non-steroidal anti-inflammatory drugs and idiopathic ulcers are rising in incidence^1,2^

In addition to classical hemostatic techniques such as clipping, application of heat, and injection therapy, hemostatic powders were developed (Hemospray, Cook Medical, Bloomington, IN, USA; EndoClot, Micro-Tech, Nanjing, China) for treatment of GI bleeding^3–6^. Hemospray (HS) is an inert bentonite powder, that does not include pharmacological or biological active compounds, but works on a more mechanic-physically basis by hygroscopicity. HS is applied endoscopically through a catheter onto the surface of the bleeding site to form a coating which prevents further bleeding or re-bleeding, and is approved in the European Union for use in the upper GI tract and in Canada for use in the upper and lower GI tract. While the classical hemostatic techniques are very effective in treating punctual or small bleeding sites, the hemostatic powders have their key advantage in the non-contact treatment of wider areas of diffuse bleeding, such as tumor infiltration, or massive ulceration. Over time, the scope of Hemospray application (HSA) was expanded to include treatment of lower gastrointestinal bleeding, although it was an off-label use at that time^7,8^.

However, the pathophysiological mechanism of action of hemostatic powders is not elucidated so far. In clinical routine we observed healing of chronic and diffuse ulcerations in some patients^9^. Literature research shows an animal study and a some case report of patients with healing ulcerations of the gastrointestinal tract following HSA^10,11^. Thus, beside the well-documented hemostatic effect, other pleiotropic beneficial effects of HSA may include acceleration of mucosal healing. Again, possible underlying mechanisms are yet unknown. In addition, directly acting of mucosa protecting therapeutics has been discussed for inflammatory bowel diseases such as ulcerative colitis for many years^12^.

The aim of this study was to evaluate a possible beneficial effect of HSA on ulcer healing in an in-vivo porcine model of upper and lower GI-ulceration and to generate hypotheses for further research.

## Results

### Changes in ulcer size

**Figure 1:**
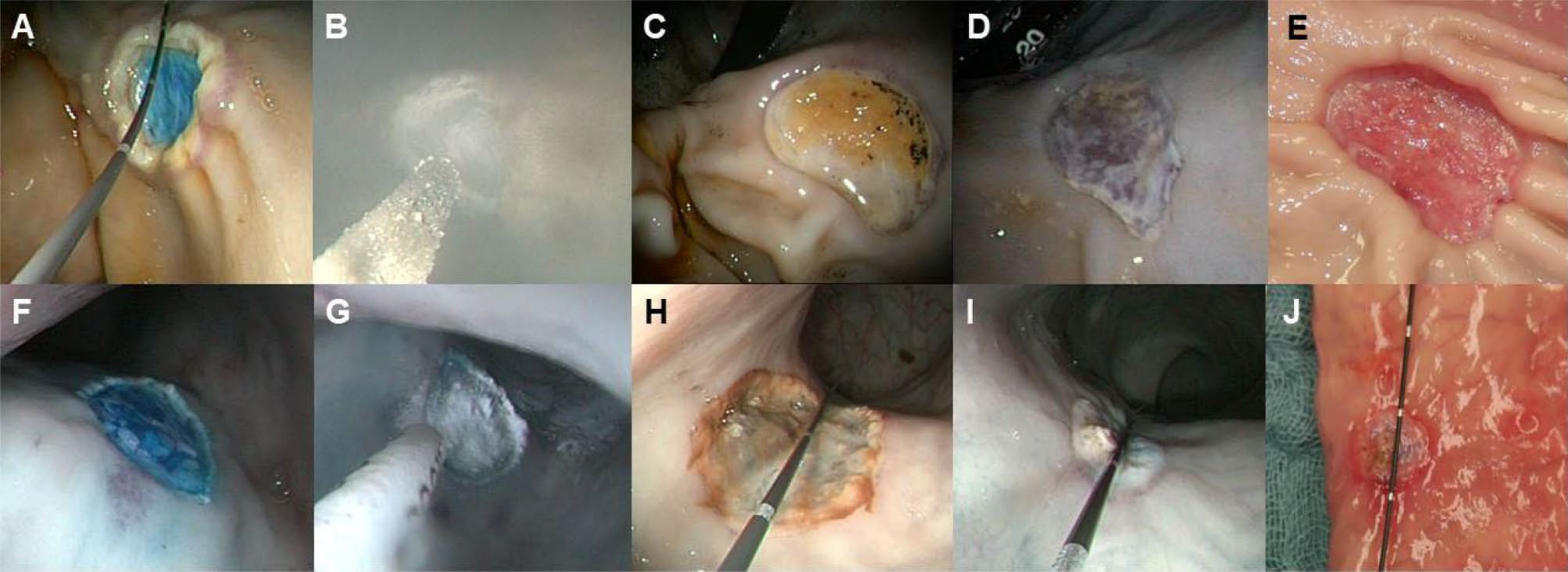
Workflow of the study in the porcine model (treatment group). Gastral lesions are shown in the upper line, rectal lesions in the lower line. (**A**, **F**) Freshly EMR-induced ulcers in the stomach and in the rectum. In (**B**, **G**) application of HS on both locations is presented. (**C**, **H**) Present the ulcers on day 2, and (**D, E, I, J**) on day 7 (end of study; (**D**, **I**)-endoscopically; (**E, J**)-ex vivo). Measurement of lesions with the centimeter-calibrated tip-wire is shown in (**A, G, H, J**).

**Figure 2:**
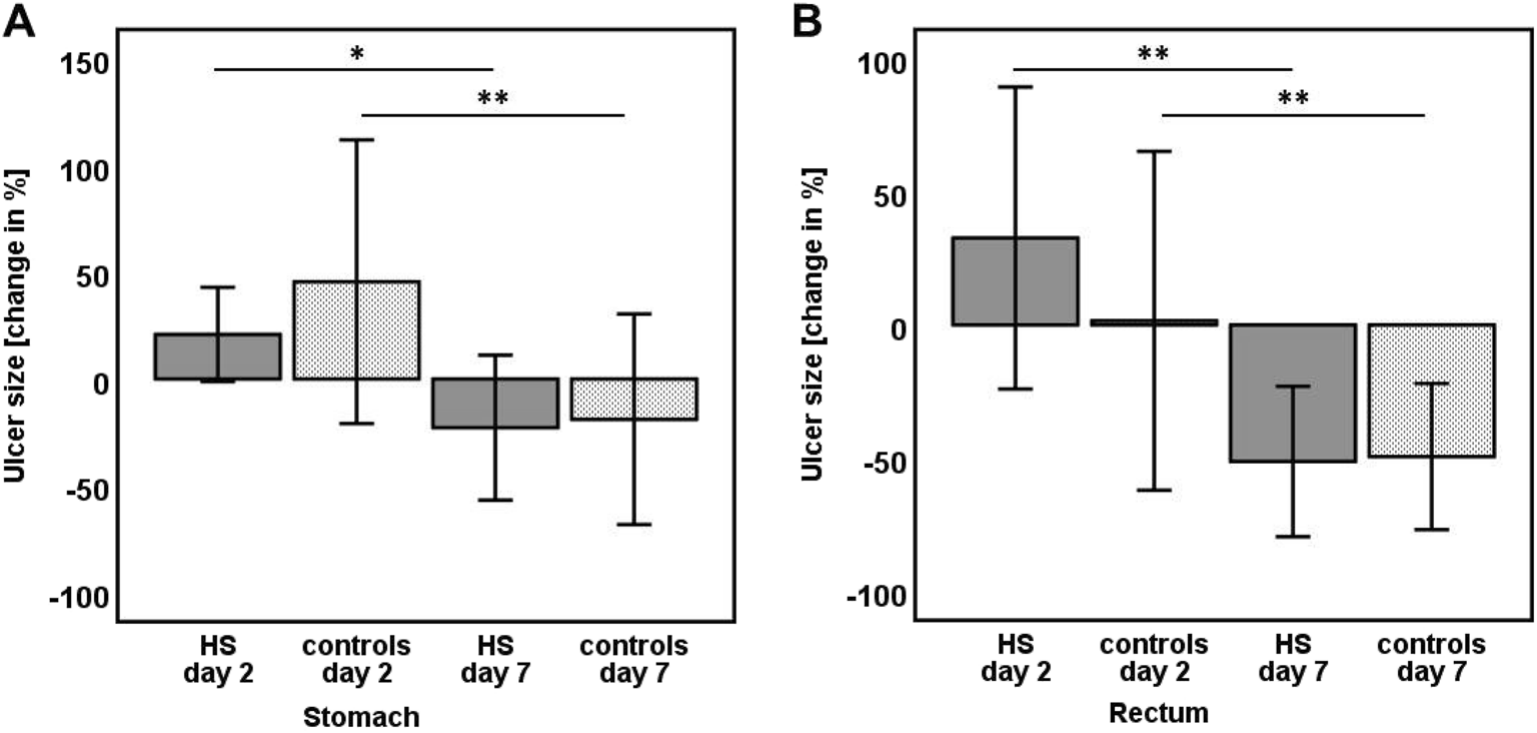
Changes in gastric and rectal ulcer size. Change in % relating to baseline, means and SD are shown. *p < 0.05, **p < 0.03, Wilcoxon’s test. Changes in % in ulcer size between the HS-treated and the non-treated group of pigs were not significant on day 2, and 7 neither in the stomach nor in the rectum. *HS* Hemospray.

By endoscopic measurement, both in the stomach and in the rectum of the pigs, the size of the ulcers increased in size from day 0 to day 2. This was most probably because of additional necrosis of cauterized tissue at the margins of the ulcers and due to the skimming of tissue crinkles after EMR (Fig. 1). Comparing day 0 with day 7, as expected, the ulcers decreased in size due to mucosal healing (stomach: − 22.8% with HSA, − 19% without HSA; rectum: − 50.8% with HSA, − 49.5% without HSA, see Tables 1, 2, and Fig. 2 for details). The decrease in ulcer size between day 0 and day 7 was significant in all groups, whether treated with HSA or not (P in the range of 0.005—0.037, Wilcoxon’s test).

**Table 1:** Comparison of changes in ulcer size after 2 and 7 days (end of study), immune-histochemical studies, and thickness of collagen layer at day 7 between HS-treated and non-treated pigs.

Ulcer size [change in % of baseline; mean (SD)]	Stomach day 2	Stomach day 7	Wilcoxon *p *value
HSA	20.8 (30.9)	− 22.8 (47.6)	**0.037**
Control	45.6 (93)	− 19 (69.1)	**0.008**

	Rectum day 2	Rectum day 7	
HSA	37.9 (79.7)	− 50.8 (39.4)	**0.005**
Control	1.5 (89.1)	− 49.5 (38.4)	**0.017**

	HSA	control	Mann–Whitney U
Stomach day 2	20.8 (30.9)	45.6 (93)	0.971
Stomach day 7	− 22.8 (47.6)	− 19 (69.1)	0.684
Rectum day 2	37.9 (79.7)	1.5 (89.1)	0.165
Rectum day 7	− 50.8 (39.4)	− 49.5 (38.4)	0.796

Immunohistochemistry at day 7 [ERG + cells/ HPF; mean (min; max)]	HSA	Control	
Stomach ulcer ground	226.7 (136.9; 316.6)	267.4 (154.1; 381.7)	0.393
Stomach ulcer margin	155.1 (97.9; 250.4)	176.7 (121.5; 236.5)	0.165
Rectum ulcer ground	237.7 (170.5; 328.5)	209 (152.3; 248)	1
Rectum ulcer margin	245.3 (132.3; 324.9)	200.6 (171.5; 249.5)	0.247

Thickness of collagen layer at day 7 [µm; mean (min; max)]	HSA	Control	
Stomach ulcer ground	1220.2 (528.8; 2355.8)	1058.1 (440.1; 1643.2)	0.853
Stomach ulcer margin	892.3 (402.6; 1539)	785.7 (510.2; 1253.8)	0.971
Rectum ulcer ground	1672.4 (1184; 1966.8)	1534.7 (926.2; 1962.3)	0.780
Rectum ulcer margin	1422.8 (1173.7; 1712.6)	1340.2 (967.1; 1709.9)	0.278

Wilcoxon’s test and Mann–Whitney U test were used for statistical analyses. Significant results are printed in bold.

*ERG* member of the ETS family of transcription factor, *HPF* high power field, *HSA* Hemospray application, *SD* standard deviation.

**Table 2:** Details of endoscopic procedures.

Variable	HAS	Control	Mann Whitney U *p *value
Ulcer size stomach at baseline [mean mm^2^ (SD)]	605 (331)	384 (252)	0.075
Ulcer size rectum at baseline [mean mm^2^ (SD)]	151 (102)	206 (69)	0.218
Major bleeding rate [n (%)]	2 (10)	0 (0)	
Perforation rate [n (%)]	0 (0)	0 (0)	

Mann–Whitney U test was used for statistical analyses.

*HSA* Hemospray application, *SD* standard deviation.

### Macroscopic effect of HSA

**Figure 3:**
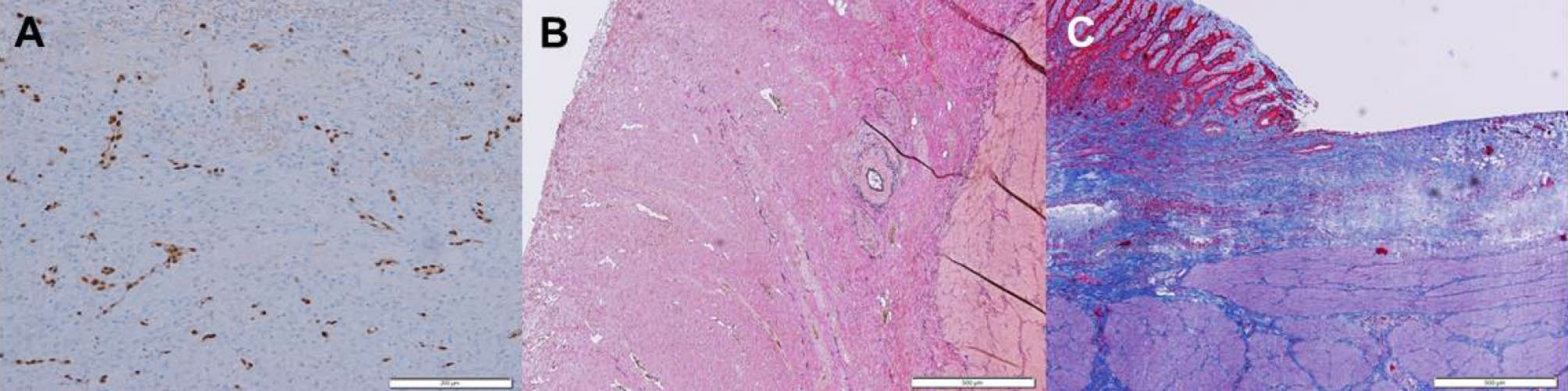
Analysis of histological parameters. (**A**) Immunohistochemistry (ERG staining of ulcer ground, white bar indicates 200 µm); (**B**) Elastica-van-Gieson staining of ulcer ground (white bar indicates 500 µm); (**C**) Masson-Trichrome staining of the ulcer margin (white bar indicates 500 µm). *ERG* member of the ETS family of transcription factor.

There were no significant differences in relative changes of ulcer sizes at day 7 between both groups of pigs, whether treated with HSA or not, neither in the stomach (*p* = 0.68; Mann–Whitney U), nor in the rectum (*p* = 0.796; Mann–Whitney U; see Table 2, and Fig. 3 for details). Interestingly, though not significant, at day two after the induction of ulcers in the stomach, the relative ulcer size in the stomach of HSA-treated pigs was smaller than in the non-treated group, whereas the size of the non-treated ulcers in the rectum was smaller than in the HSA treated group. Additionally, though not significant, in the rectum, the relative decrease of the ulcer size from day 2 to day 7 in the HSA treated group exceeded the relative decrease in ulcer size of the non-treatment group (− 84.1% with HS, − 51% without HS).

### Histological effect of HSA

Expression of ERG on both the ulcer ground and margin (see Table 2 and Fig. 2 for details) was quantified. In our study, there were no significant differences in the amount of ERG-positive endothelial cells between the group of pigs, which were treated with HS, and those, which were not treated with HSA (*p* = 0.123–*p* = 0.436; Mann–Whitney U). Additionally, we measured the thickness of the collagen layer of the ulcers at day seven. There was also no significant difference between the thickness of the collagen layers between HSA treated animals and controls (*p* = 0.496–*p* = 0.705, Mann–Whitney U). Of note, in rectal ulcers and stomach ulcers of HSA treated pigs, there was a trend towards a thicker layer of collagen fibers, which could possibly suggest a more intensive activity of regeneration.

### Detection of Hemospray components in the ulcers

On histological examination at day 7 optically refractive crystals, which could be remnants of the HSA, were not observed in any of 20 HSA treated ulcers.

### Side effects

In one pig, arterial bleeding of both stomach ulcers occurred during EMR, which was immediately treated by endoscopic clipping. The pig was additionally treated by HSA. Hemoglobin values were stable in this pig afterwards, as they were in all other pigs.

## Discussion

HSA is approved and widely used for the treatment of bleeding in the upper GI tract^1,2^, but to our knowledge pathophysiological effects on mucosal healing in the upper and lower GI tract have not been studied so far in vivo on a longitudinal endoscopic and histologic basis.

In our study we induced mucosal lesions in the stomach and rectum of pigs. Half of the pigs were treated by HSA after induction of the lesions. The other half of pigs was the control group without HSA after induced ulcers. On endoscopic examination, the relative decrease in ulcer size over time was similar in both groups (see Table 2). Interestingly, the ulcers in the lower GI-tract seemed to heal in a faster fashion than the ulcers in the upper GI-tract. However, in this localization, just as in the upper GI tract, no significant difference between both groups of pigs, the HS treated and the untreated, could be observed.

In our histological studies, no significant effect of HSA on ulcer healing could be detected with respect to neo-angiogenesis (ERG positive endothelial cells) assays, and to regeneration (thickness of collagenous layer), though a non-significant trend towards thicker collagenous layers in HS treated ulcers was observed.

Our study was designed as a pilot study to generate hypotheses for further studies, and has inherent strengths and weaknesses: some observations indicated a potential mechanism for HSA on mucosal healing beyond the mere hemostatic effect, though none of these reached the level of significance in our exploratory statistical analysis: In the stomach, at day 2 after induction of the ulcers, the mean relative size of the ulcers in the HSA treated group of pigs was smaller than in the group without treatment. This could be due to a short-termed protective effect of HSA on the ulcers, Accordingly, in the rectum, although not significant possibly due to the small number of pigs, the decrease of the relative sizes of the ulcers from day 2 to day 7 in the HSA treatment group exceeded that of the group of non-treated pigs, which could be an expression of a medium-term protective effect. Additionally, a tendency for a thicker collagenous layer in the HSA treated pigs was noticed, thus possibly suggesting an effect on deeper areas of the GI-ulcers, even though not significant.

We could show that no trace of HSA was seen in the microscopic evaluation of the lesions. It points to an early differential induction in ulcer healing with potential effects even after the mechanical barrier formed by HSA has worn off.

Given the low number of animals and the well documented clinical experience with HSA in high patient numbers^13^, we did not expect to unravel a significant effect that had not been observed before. However, our findings, if confirmed in further studies, may point towards a mucosa-protective effect apart from the mechanical hemostatic efficacy. In clinical practice, this could help the managing physician by creating a short “window of opportunity” to treat underlying diseases such as starting anti-inflammatory treatment in inflammatory bowel disease or withdrawing non-steroidal anti-inflammatory drugs. This potential application is supported by case reports^9,14^.

Therefore, it is of interest whether the HSA for hemostasis has adverse effects on the short-term healing of mucosal lesions, thus possibly jeopardizing the intended effects of the endoscopic treatment in the presence of alternative treatment modalities like clipping, Argon Plasma Coagulation and localized cauterization. We could not document any unfavorable effect on ulcer healing in the upper and lower GI tract, with respect to endoscopic decrease of ulcer size and histological parameters.

In summary, our pilot study shows no significant effect of HSA on ulcer healing in the upper and lower GI tract compared with untreated controls.

## Methods

### Design

This animal study used in-vivo interventions and examinations to compare the performance of HSA in fresh ulcerations in the gastric and rectal position. In sum ten domestic female pigs weighing about 40 kg (mean 40.9 kg ± 3.2) were examined. In each pig two gastric and rectal ulcerations were created with an endoscopic mucosal resection (EMR). Five pigs were treated with HSA on the fresh ulceration; five pigs were not treated as control group. This was performed to avoid “cross-contamination” of lower GI lesions by application of HS in the stomach. Endoscopic follow-up examinations were performed two days and seven days after the initial EMR. Primary endpoint of the study was the change in size of the ulcers after 7 days post treatment. Secondary endpoints were differences in angiogenetic markers between both groups, and differences in the thickness of collagen layers after 7 days post treatment.

### Sample size

Since there was no previous experience in the use of HSA for the healing of mucosal lesions, a size reduction of 25% after 7 days was assumed. A case number of 20 ulcerations in the upper and 20 ulcerations in the lower GI tract were assumed sufficient for detecting differences between both groups.

### Ethical and regulatory background

The protocol of this study was approved by the local institutional review board (Regierungspräsidium Tübingen, Aktenzeichen 35/9185.81-2/Tierversuch-Nr. M 10/15, 02.09.2015), all methods are reported in accordance with ARRIVE guidelines.

### Implementation of the examinations

24 h before gastric and rectal EMR the pigs received liquid food. In each pig, two EMR ulcers were created in the stomach and in the rectum, respectively, under general anesthesia. 5 of the pigs were treated with local HSA immediately after EMR on all locations, and 5 of the pigs were not. All pigs were treated with proton-pump inhibitors (pantoprazole) 40 mg twice a day until the end of the study. The EMR lesions were measured endoscopically at baseline, day 2, and day 7 using an endoscopic wire with a centimeter-calibrated tip. At day 7 the pigs were sacrificed. The EMR specimens at baseline and the full thickness of the remaining ulcers at day 7 were obtained for histopathological analyses. All steps were documented photographically (Fig. 1).

### Endoscopic procedures

A conventional endoscope (13801PKS-X, Karl Storz Endoskope, Tuttlingen, Germany) was used. EMR were performed using a disposable 23-gauge injection needle (medwork, Höchstadt/Aich, Germany), a 25 mm asymmetric snare (Acusnare, Cook Medical, Bloomington, IN, USA), and an electrosurgical unit (VIO 300D, Erbe Elektromedizin, Tübingen, Germany). For EMR, 0.9% saline mixed with adrenaline 1:100,000 and methylene blue was injected into the submucosal layer of the antrum of the stomach, forming a cushion, which then was dissected with the electrosurgical snare. The same procedure was repeated at the corpus of the stomach (Table 1, and Fig. 1). After that, in half of the pig’s HSA was sprayed onto the lesions and the surrounding intact mucosa according to manufacturer’s guidance. The same procedures were performed in the rectum of the pigs, and likewise, in half of the pigs HS was applied to the surface of the lesions and intact mucosa (Fig. 1). On the seventh day, the pigs were sacrificed. The organs were harvested for ex vivo measurement of the sizes of the lesions. Ulcers were prepared for pathological examination (Fig. 1).

### Measurement of ulcer size

For endoscopic assessment of the size of a lesion in vivo in a tubular organ, a centimeter-calibrated tipped endoscopic wire (Acrobat 0.035″, Cook Medical, Bloomington, IN, USA; see Fig. 1) was used. The area of the lesions was then calculated with the ellipsoid-formula. All measurements from photo or video documentation were performed in duplicate by two single blinded investigators (CRW, LB). On the second and seventh day the sizes of the lesions in the stomach and in the rectum of the pigs were measured again with the calibrated wire.

### Histopathological assessment

Pathological specimens were stained with hematoxylin and eosin for light microscopy.

Additionally, the specimens were stained with the immune-histochemical (IHC) marker ERG (member of the ETS family of transcription factor), an endothelial marker for identification of the capillary ingrowth into the ulcer ground and margins^15–18^. For quantification of that process, the IHC positive cells were counted computer-aided (ImageJ, Wayne Rasband, Research Services Branch, National Institute of Mental Health, Bethesda, Maryland, USA; Fig. 2).

For assessment of the progress of the healing process of the ulcers, the thickness of the collagen fiber layer at the ground and on the margins of the ulcers were measured, assuming a positive association between thickness of collagenous layer and better ulcer healing. For this, the tissue sections were stained with Elastica-van-Gieson and Masson-Trichrome (see Fig. 2).

### Statistical analyses

Descriptive statistics: Continuous data are expressed as the mean and confidence intervals 5–95%, or, if meaningful, as median values. Means between groups were compared by the Mann–Whitney *U* test performed in SPSS software v. 25 (IBM Corp., Armonk, NY, USA). Standard deviation (SD) is stated.

## Data Availability

The datasets used and/or analysed during the current study are available from the corresponding author on reasonable request.
